# Adapting Laplace residual power series approach to the Caudrey Dodd Gibbon equation

**DOI:** 10.1038/s41598-024-57780-x

**Published:** 2024-04-29

**Authors:** Samy A. Abdelhafeez, Anas A. M. Arafa, Yousef H. Zahran, Ibrahim S. I. Osman, Moutaz Ramadan

**Affiliations:** 1 Department of Mathematics and Computer Science, Faculty of Science, Port Said University, Port Said, Egypt; 2 Department of Mathematics, College of Science, Qassim University, Buraydah, Saudi Arabia; 3 Physics and Mathematics Department, Faculty of Engineering, Port Said University, Port Said, Egypt

**Keywords:** Fractional derivatives, Laplace transform, Residual power series method, Caudrey Dodd Gibbon equation, Numerical results, Engineering, Mathematics and computing

## Abstract

In real-life applications, nonlinear differential equations play an essential role in representing many phenomena. One well-known nonlinear differential equation that helps describe and explain many chemicals, physical, and biological processes is the Caudrey Dodd Gibbon equation (CDGE). In this paper, we propose the Laplace residual power series method to solve fractional CDGE. The use of terms that involve fractional derivatives leads to a higher degree of freedom, making them more realistic than those equations that involve the derivation of an integer order. The proposed method gives an easy and faster solution in the form of fast convergence. Using the limit theorem of evaluation, the experimental part presents the results and graphs obtained at several values of the fractional derivative order.

## Introduction

Calculus is one of the branches of great importance, especially differential equations of various types, whether ordinary or partial. Fractional differential equations have recently emerged in many applications such as plasma physics, image processing, laser optics, biomedical engineering, viscoelasticity, hydrology, signal processing and control system^1–13^. Some of these equations do not have an analytical solution, so we resort to approximate solutions using distinct analytical methods such as: the adomain decomposition method^14^, the variational iteration method^15^, the homotopy method^16–18^, and the Gegenbauer wavelet method^19^. In recent years, homeopathic techniques have been combined with integral transformations and dealing with different types of mathematical models by several authors^20–28^. In this paper, we will study the behavioral solution for fractional CDGE, which takes the following form:

1 $$D_{t}^{\alpha } {\text{u}} + u_{xxxxx} + 30uu_{xxx} + 30u_{x} u_{xx} + 180u^{2} u_{x} = 0,$$

where $D_{t}^{\alpha }$ denote the fractional derivatives of Caputo sense^29^. The advantage of using Caputo's derivative is that it has the memory of nonlinear partial differential equations which occurs in the physical problems. It also uses the initial conditions found in classical differential equations. For $\alpha =1$, Eq. (1) represents the classical CDGE introduced by Caudery, Dodd and Gibbon^30^. The equation is studied as a mathematical model for internal waves in shallow waters of small amplitude and long wavelength. It is also a very important phenomenon in plasma and laser physics. CDGE solutions have been presented by many mathematicians^31–39^. These methods have their drawbacks, limitations, huge computational work, larger computer memory and time, and varying results. The novelty of the results lies in obtaining new solutions easily, quickly, and with high accuracy using a LRPS method that enables us to study the CDG equation better.

This article begins with introduction which include brave history of fractional calculus. Section “Preliminaries” give some definitions and mathematical premises necessary for the theory of fractional theory. In Section “Constructing the LRPSM for the CDGE”, we show the steps of LRPS for solving the fractional CDGE. In Section “Numerical examples”, Numerical results are presented. Discussions and Conclusion are presented in Section “Discussions and conclusion”.

## Preliminaries

In this section, we will review some definitions of fractional calculus.

### Definition 1

The Riemann–Liouville fractional integral of order $\alpha$ is given as^4^

$$\begin{aligned} J^{\alpha } f\left( x \right) & = { }\frac{1}{{{\Gamma }\left( \alpha \right)}}\mathop \smallint \limits_{0}^{x} \left( {x - t} \right)^{\alpha - 1} f\left( t \right)dt,{ }\alpha > 0,{ }x > 0, \\ J^{0} f\left( x \right) & = f\left( x \right). \\ \end{aligned}$$

### Definition 2

The ${\alpha }^{th}$ order Caputo time fractional derivative of $u\left(x,t\right)$ is defined as^4^

$$D_{t}^{\alpha } { }u\left( {x,t} \right) = \left\{ {\begin{array}{*{20}l} {\frac{1}{{{\Gamma }\left( {m - \alpha } \right)}}\mathop \smallint \limits_{0}^{t} \left( {t - \zeta } \right)^{m - \alpha - 1} \frac{{\partial^{m} u\left( {x,\zeta } \right)}}{{\partial t^{m} }}d\zeta ,} \hfill & {m - 1{ } < \alpha < m,} \hfill \\ {\frac{{\partial^{m} u\left( {x,\zeta } \right)}}{{\partial t^{m} }},} \hfill & {\alpha = m \in N.} \hfill \\ \end{array} } \right.$$

### Definition 3

The Laplace transform of Caputo time fractional derivative is defined as:

$${\mathcal{L}}\left\{ {D_{t}^{\alpha } f\left( {x,t} \right)} \right\} = { }\frac{{s^{m} F\left( {x,s} \right) - { }s^{m - 1} {\text{f}}\left( {{\text{x}},0} \right){ } - { }s^{m - 2} f^{\prime}\left( {{\text{x}},0} \right){ } - { }s^{m - 3} f^{\prime\prime}\left( {{\text{x}},0} \right){ } - \cdots - { }f^{m - 1} \left( {{\text{x}},0} \right)}}{{s^{m - \alpha } }}.$$

$${\mathcal{L}}\left\{ {D_{t}^{\alpha } f\left( {x,t} \right)} \right\} = s^{\alpha } F\left( {x,s} \right) - \mathop \sum \limits_{j = 0}^{m - 1} s^{\alpha - j - 1} f_{t}^{\left( j \right)} \left( {x,0} \right),\;m - 1 < \alpha \le m,\;m \in N.$$

More details using Laplace transform found in^20–24^.

### Theorem 1

^36^. If $U\left(x,s\right)=L[u(x,t]$ contains multiple fractional power series which is define as:

$U\left(x,s\right)$=$\sum_{n=0}^{\infty }\sum_{k=0}^{m-1}\frac{{f}_{nk}\left(x\right)}{{s}^{k+n\alpha +1}}$, $0\le m-1<\alpha \le m$, then the coefficients, ${f}_{nk}\left(x\right)$ take the form:

$$f_{nk} \left( x \right) = \left\{ {\begin{array}{*{20}l} {D_{t}^{k} u\left( {x,0} \right),} \hfill & {k = 0,1, \ldots ,m - 1} \hfill \\ {D_{t}^{k} D_{t}^{n\alpha } u\left( {x,0} \right),} \hfill & {k = 0,1, \ldots ,m - 1, n = 1,2, \ldots } \hfill \\ \end{array} } \right\}.$$

### Proof

See^40^.

## Constructing the LRPSM for the CDGE

In this section, we show the steps of using LRPSM for solving the fractional CDGE.

Consider a Caputo fractional CDGE in the operator form:

2 $${D}_{t}^{\alpha }{\text{u}}\left({\text{x}},{\text{t}}\right)+ {D}_{x}^{5}{\text{u}}\left({\text{x}},{\text{t}}\right)+30{\text{u}}\left({\text{x}},{\text{t}}\right){D}_{x}^{3}{\text{u}}\left({\text{x}},{\text{t}}\right)+30{D}_{x}{\text{u}}\left({\text{x}},{\text{t}}\right){D}_{x}^{2}{\text{u}}\left({\text{x}},{\text{t}}\right)+180{u}^{2}\left(x,t\right){D}_{x}{\text{u}}\left({\text{x}},{\text{t}}\right)=0,$$

for t $$>0$$, $$x\in R$$, $$m-1<\alpha <m.$$

The main idea of LRPSM in few steps as follow:

*Step 1.* Apply the Laplace transform to Eq. (2) as:

3 $$\mathcal{L}\{{D}_{t}^{\alpha }{\text{u}}({\text{x}},{\text{t}})+ {D}_{x}^{5}{\text{u}}({\text{x}},{\text{t}})+30{\text{u}}({\text{x}},{\text{t}}) {D}_{x}^{3}{\text{u}}({\text{x}},{\text{t}})+30{D}_{x}{\text{u}}({\text{x}},{\text{t}}) {D}_{x}^{2}{\text{u}}({\text{x}},{\text{t}})+180{u}^{2}(x,t){D}_{x}{\text{u}}({\text{x}},{\text{t}})\} =0.$$

Then we obtained

4 $$\frac{{\text{sU}}\left({\text{x}},\mathrm{ s}\right)-{\text{u}}({\text{x}},0)}{{s}^{1-\alpha }}+{D}_{x}^{5}{\text{U}}({\text{x}},{\text{s}})+30\mathcal{L}\{{\mathcal{L}}^{-1}\left({\text{U}}\left({\text{x}},{\text{s}}\right)\right){\mathcal{L}}^{-1}\left({D}_{x}^{3}{\text{U}}\left({\text{x}},{\text{s}}\right)\right)\}+30\mathcal{L}\{{\mathcal{L}}^{-1}\left({D}_{x}{\text{U}}\left({\text{x}},{\text{s}}\right)\right){\mathcal{L}}^{-1}\left({D}_{x}^{2}{\text{U}}\left({\text{x}},{\text{s}}\right)\right)\}+180{\mathcal{L}\left\{{(\mathcal{L}}^{-1}U\left(x,s\right)\right)}^{2}{{(\mathcal{L}}^{-1}D}_{x}{\text{U}}({\text{x}},{\text{s}}))\} =0.$$

Multiply Eq. (4) by ${s}^{-\alpha }$ we get

5 $$U\left( {x,s} \right) - \frac{{u\left( {x,0} \right)}}{s} + \frac{1}{{s^{\alpha } }}D_{x}^{5} {\text{U}}\left( {{\text{x}},{\text{s}}} \right) + \frac{30}{{s^{\alpha } }}{\mathcal{L}}\left\{ {{\mathcal{L}}^{ - 1} \left( {{\text{U}}\left( {{\text{x}},{\text{s}}} \right)} \right){\mathcal{L}}^{ - 1} \left( {D_{x}^{3} {\text{U}}\left( {{\text{x}},{\text{s}}} \right)} \right)} \right\} + \frac{30}{{s^{\alpha } }}{\mathcal{L}}\left\{ {{\mathcal{L}}^{ - 1} \left( {D_{x} {\text{U}}\left( {{\text{x}},{\text{s}}} \right)} \right){\mathcal{L}}^{ - 1} \left( {D_{x}^{2} {\text{U}}\left( {{\text{x}},{\text{s}}} \right)} \right)} \right\} + \frac{180}{{s^{\alpha } }}{\mathcal{L}}\left\{ {\left( {{\mathcal{L}}^{ - 1} U\left( {x,s} \right)} \right)^{2} \left( {{\mathcal{L}}^{ - 1} D_{x} {\text{U}}\left( {{\text{x}},{\text{s}}} \right)} \right)} \right\}{ } = 0.$$

*Step 2*. We can write the transformed function $U(x,s)$ as the following expansion

6 $$U\left( {x,s} \right) = \mathop \sum \limits_{n = 1}^{\infty } \frac{{f_{n} \left( x \right)}}{{s^{n\alpha + 1} }}.$$

The kth-truncated series (6) take the form:

7 $$U_{k} \left( {x,s} \right) = \mathop \sum \limits_{n = 1}^{k} \frac{{f_{n} \left( x \right)}}{{s^{n\alpha + 1} }} = \frac{{f_{0} \left( x \right)}}{s} + \mathop \sum \limits_{n = 1}^{k} \frac{{f_{n} \left( x \right)}}{{s^{n\alpha + 1} }}.$$

The Laplace residual function define as:

8 $$\begin{aligned} {\mathcal{L}}Res\left( {x ,s} \right) & = U\left( {x,s} \right) - \frac{{u\left( {x,0} \right)}}{s} + \frac{1}{{s^{\alpha } }}D_{x}^{5} {\text{U}}\left( {{\text{x}},{\text{s}}} \right) + \frac{30}{{s^{\alpha } }}{\mathcal{L}}\left\{ {{\mathcal{L}}^{ - 1} \left( {{\text{U}}\left( {{\text{x}},{\text{s}}} \right)} \right){\mathcal{L}}^{ - 1} \left( {D_{x}^{3} {\text{U}}\left( {{\text{x}},{\text{s}}} \right)} \right)} \right\} \\ & \quad + \frac{30}{{s^{\alpha } }}{\mathcal{L}}\left\{ {{\mathcal{L}}^{ - 1} \left( {D_{x} {\text{U}}\left( {{\text{x}},{\text{s}}} \right)} \right){\mathcal{L}}^{ - 1} \left( {D_{x}^{2} {\text{U}}\left( {{\text{x}},{\text{s}}} \right)} \right)} \right\} + \frac{180}{{s^{\alpha } }}{\mathcal{L}}\left\{ {\left( {{\mathcal{L}}^{ - 1} U\left( {x,s} \right)} \right)^{2} \left( {{\mathcal{L}}^{ - 1} D_{x} {\text{U}}\left( {{\text{x}},{\text{s}}} \right)} \right)} \right\}. \\ \end{aligned}$$

The kth-Laplace residual function defines as:

9 $$\begin{aligned} {\mathcal{L}}Res_{k} \left( {x,s} \right) & = U_{k} \left( {x,s} \right) - \frac{{u\left( {x,0} \right)}}{s} + \frac{1}{{s^{\alpha } }}D_{x}^{5} U_{k} \left( {x,s} \right) + \frac{30}{{s^{\alpha } }}{\mathcal{L}}\left\{ {{\mathcal{L}}^{ - 1} \left( {U_{k} \left( {x,s} \right)} \right){\mathcal{L}}^{ - 1} \left( {D_{x}^{3} U_{k} \left( {x,s} \right)} \right)} \right\} \\ & \quad + \frac{30}{{s^{\alpha } }}{\mathcal{L}}\left\{ {{\mathcal{L}}^{ - 1} \left( {D_{x} U_{k} \left( {x,s} \right)} \right){\mathcal{L}}^{ - 1} \left( {D_{x}^{2} U_{k} \left( {x,s} \right)} \right)} \right\} + \frac{180}{{s^{\alpha } }}{\mathcal{L}}\left\{ {\left( {{\mathcal{L}}^{ - 1} U_{k} \left( {x,s} \right)} \right)^{2} \left( {{\mathcal{L}}^{ - 1} D_{x} U_{k} \left( {x,s} \right)} \right)} \right\}. \\ \end{aligned}$$

To determine the coefficient function $f_{n} \left( x \right)$, we substitute the kth-truncated series (7) into Eq. (9), multiply the resulting equation by $s^{k\alpha + 1}$ and then solve recursively the following system:

10 $$\mathop {\lim }\limits_{s \to \infty } s^{k\alpha + 1} {\mathcal{L}}Res_{k} \left( s \right) = 0\;{\text{where}}\;{\text{k}} = {1},{2},{ 3} \ldots$$

Then we have

11 $${s}^{k\alpha +1}\mathcal{L}{Res}_{k}\left(x,s\right)={s}^{k\alpha +1}\sum_{n=1}^{k}\frac{{f}_{n}\left(x\right)}{{s}^{n\alpha +1}}+\frac{{s}^{k\alpha +1}}{{s}^{\alpha }}{D}_{x}^{5}(\frac{{f}_{0}\left(x\right)}{s}+\sum_{n=1}^{k}\frac{{f}_{n}\left(x\right)}{{s}^{n\alpha +1}})+\frac{{s}^{k\alpha +1}}{{s}^{\alpha }}\mathcal{L}\left\{{\mathcal{L}}^{-1}\left(\frac{{f}_{0}\left(x\right)}{s}+\sum_{n=1}^{k}\frac{{f}_{n}\left(x\right)}{{s}^{n\alpha +1}}){\mathcal{L}}^{-1}\left({D}_{x}^{3}\left(\frac{{f}_{0}\left(x\right)}{s}+\sum_{n=1}^{k}\frac{{f}_{n}\left(x\right)}{{s}^{n\alpha +1}}\right)\right)\right\}+\frac{{s}^{k\alpha +1}}{{s}^{\alpha }}\mathcal{L}\left\{{\mathcal{L}}^{-1}\left({D}_{x}(\frac{{f}_{0}\left(x\right)}{s}+\sum_{n=1}^{k}\frac{{f}_{n}\left(x\right)}{{s}^{n\alpha +1}})\right){\mathcal{L}}^{-1}\left({D}_{x}^{2}(\frac{{f}_{0}\left(x\right)}{s}+\sum_{n=1}^{k}\frac{{f}_{n}\left(x\right)}{{s}^{n\alpha +1}})\right)\right\}+\frac{{s}^{k\alpha +1}}{{s}^{\alpha }}{\mathcal{L}\left\{{(\mathcal{L}}^{-1}(\frac{{f}_{0}\left(x\right)}{s}+\sum_{n=1}^{k}\frac{{f}_{n}\left(x\right)}{{s}^{n\alpha +1}})\right)}^{2}{{(\mathcal{L}}^{-1}D}_{x}\left(\frac{{f}_{0}\left(x\right)}{s}+\sum_{n=1}^{k}\frac{{f}_{n}\left(x\right)}{{s}^{n\alpha +1}})\right)\right\}.$$

Now, to determine the coefficient function ${f}_{1}\left(x\right)$, we substitute k = 1 into Eq. (11) and hence we will obtain the relationship:

$${\underset{s\to \infty }{{\text{lim}}}{s}^{\alpha +1}\mathcal{L}{Res}_{1}\left(s\right)=s}^{\alpha +1}\frac{{f}_{1}\left(x\right)}{{s}^{\alpha +1}}+\frac{{s}^{\alpha +1}}{{s}^{\alpha }}{D}_{x}^{5}(\frac{{f}_{0}\left(x\right)}{s}+\frac{{f}_{1}\left(x\right)}{{s}^{\alpha +1}})+\frac{{s}^{\alpha +1}}{{s}^{\alpha }}\mathcal{L}\{{\mathcal{L}}^{-1}(\frac{{f}_{0}\left(x\right)}{s}+\frac{{f}_{1}\left(x\right)}{{s}^{\alpha +1}}){\mathcal{L}}^{-1}\left({D}_{x}^{3}\left(\frac{{f}_{0}\left(x\right)}{s}+\frac{{f}_{1}\left(x\right)}{{s}^{\alpha +1}}\right)\right)\}+\frac{{s}^{\alpha +1}}{{s}^{\alpha }}\mathcal{L}\{{\mathcal{L}}^{-1}\left({D}_{x}(\frac{{f}_{0}\left(x\right)}{s}+\frac{{f}_{1}\left(x\right)}{{s}^{\alpha +1}}\right){\mathcal{L}}^{-1}\left({D}_{x}^{2}(\frac{{f}_{0}\left(x\right)}{s}+\frac{{f}_{1}\left(x\right)}{{s}^{\alpha +1}}\right)\}+\frac{{s}^{\alpha +1}}{{s}^{\alpha }}{\mathcal{L}\left\{{(\mathcal{L}}^{-1}(\frac{{f}_{0}\left(x\right)}{s}+\frac{{f}_{1}\left(x\right)}{{s}^{\alpha +1}}\right)}^{2}{{(\mathcal{L}}^{-1}D}_{x}(\frac{{f}_{0}\left(x\right)}{s}+\frac{{f}_{1}\left(x\right)}{{s}^{\alpha +1}})\}.$$

By using Eq. (11), we obtained

12 $$f_{1} \left( x \right) = - \left\{ { f_{0}^{\left( 5 \right)} \left( x \right) + 30f_{0} \left( x \right)f_{0}^{\left( 3 \right)} \left( x \right) + 30f_{0}^{\left( 1 \right)} \left( x \right)f_{0}^{\left( 2 \right)} \left( x \right) + 180 f_{0}^{2} \left( x \right)f_{0}^{\left( 1 \right)} \left( x \right)} \right\}.$$

to determine the coefficient function ${f}_{2}\left(x\right)$, we substitute k = 2 into Eq. (11) and hence we will obtain the relationship:

13 $$\begin{aligned} \mathop {\lim }\limits_{s \to \infty } s^{2\alpha + 1} {\mathcal{L}}Res_{2} \left( s \right) & = s^{2\alpha + 1} \left( {\frac{{f_{1} \left( x \right)}}{{s^{\alpha + 1} }} + \frac{{f_{2} \left( x \right)}}{{s^{2\alpha + 1} }}} \right) + \frac{{s^{2\alpha + 1} }}{{s^{\alpha } }}D_{x}^{5} \left( {\frac{{f_{0} \left( x \right)}}{s} + \frac{{f_{1} \left( x \right)}}{{s^{\alpha + 1} }} + \frac{{f_{2} \left( x \right)}}{{s^{2\alpha + 1} }}} \right) \\ & \quad + \frac{{s^{2\alpha + 1} }}{{s^{\alpha } }}{\mathcal{L}}\left\{ {{\mathcal{L}}^{ - 1} \left( {\frac{{f_{0} \left( x \right)}}{s} + \frac{{f_{1} \left( x \right)}}{{s^{\alpha + 1} }} + \frac{{f_{2} \left( x \right)}}{{s^{2\alpha + 1} }}} \right){\mathcal{L}}^{ - 1} \left( {D_{x}^{3} \left( {\frac{{f_{0} \left( x \right)}}{s} + \frac{{f_{1} \left( x \right)}}{{s^{\alpha + 1} }} + \frac{{f_{2} \left( x \right)}}{{s^{2\alpha + 1} }} } \right)} \right)} \right\} \\ & \quad + \frac{{s^{2\alpha + 1} }}{{s^{\alpha } }}{\mathcal{L}}\left\{ {{\mathcal{L}}^{ - 1} \left( {D_{x} \left( {\frac{{f_{0} \left( x \right)}}{s} + \frac{{f_{1} \left( x \right)}}{{s^{\alpha + 1} }} + \frac{{f_{2} \left( x \right)}}{{s^{2\alpha + 1} }}} \right)} \right){\mathcal{L}}^{ - 1} \left( {D_{x}^{2} \left( {\frac{{f_{0} \left( x \right)}}{s} + \frac{{f_{1} \left( x \right)}}{{s^{\alpha + 1} }} + \frac{{f_{2} \left( x \right)}}{{s^{2\alpha + 1} }}} \right)} \right)} \right\} \\ & \quad + \frac{{s^{2\alpha + 1} }}{{s^{\alpha } }}{\mathcal{L}}\left\{ {{\mathcal{L}}^{ - 1} \left( {\frac{{f_{0} \left( x \right)}}{s} + \frac{{f_{1} \left( x \right)}}{{s^{\alpha + 1} }} + \frac{{f_{2} \left( x \right)}}{{s^{2\alpha + 1} }}} \right)^{2} {\mathcal{L}}^{ - 1} D_{x} \left( {\frac{{f_{0} \left( x \right)}}{s} + \frac{{f_{1} \left( x \right)}}{{s^{\alpha + 1} }} + { }\frac{{f_{2} \left( x \right)}}{{s^{2\alpha + 1} }}} \right)} \right\}. \\ \end{aligned}$$

By using Eq. (11), we obtained

14 $$f_{2} \left( x \right) = - \left\{ {f_{1}^{\left( 5 \right)} \left( x \right) + 30f_{0} \left( x \right)f_{1}^{\left( 3 \right)} \left( x \right) + 30f_{1} \left( x \right)f_{0}^{\left( 3 \right)} \left( x \right) + 30f_{0}^{\left( 1 \right)} \left( x \right)f_{1}^{\left( 2 \right)} \left( x \right) + 30f_{1}^{\left( 1 \right)} \left( x \right)f_{0}^{\left( 2 \right)} \left( x \right) + 180 f_{0}^{2} \left( x \right)f_{1}^{\left( 1 \right)} \left( x \right) + 360f_{0} \left( x \right)f_{0}^{\left( 1 \right)} \left( x \right)f_{1} \left( x \right)} \right\}.$$

And so on, we can get more coefficient function ${f}_{n}\left(x\right)$ by using Eq. (10), Eq. (11) and substitute it into Eq. (7).

*Finally*: Apply the inverse Laplace transform to ${U}_{k}\left(x,s\right)$ to obtain the kth—approximate solution ${u}_{k}\left(x,t\right).$

## Numerical examples

### Example 1

Consider the time fractional CDGE (1) with initial condition

15 $$u\left( {x,0} \right) = \frac{1}{4}k^{2} sech^{2} \left( {\frac{1}{2}kx + c} \right).$$

The exact solution in classical case is

16 $$u\left( {x,t} \right) = \frac{1}{4}k^{2} sech^{2} \left( {\frac{1}{2}kx - \frac{1}{2}k^{2} t + c} \right).$$

By using initial condition (15) and applying the steps of using LRPSM for solving the fractional CDGE which discussed in Section “Preliminaries”, we obtain

17 $$f_{0} \left( x \right) = u\left( {x,0} \right) = \frac{1}{16}sech^{2} \left( {\frac{1}{4}x + 0.5} \right),$$

18 $$f_{1} \left( x \right) = \frac{1}{512} \left( {\frac{{\sinh \left( {\frac{x}{4} + 0.5} \right)}}{{cosh^{2} \left( {\frac{x}{4} + 0.5} \right)}}} \right),$$

19 $$f_{2} \left( x \right) = \frac{1}{32768}\left( {sech^{4} \left( {\frac{x}{4} + 0.5} \right)\left\{ {\cosh \left( {\frac{x}{2} + 1} \right) - 2} \right\}{ }} \right).$$

And the approximate solution fractional CDGE is:

20 $$\begin{aligned} u\left( {x,t} \right) & = \frac{1}{16}sech^{2} \left( {\frac{x}{4} + 0.5} \right) + \frac{1}{512} \left( {\frac{{\sinh \left( {\frac{x}{4} + 0.5} \right)}}{{cosh^{2} \left( {\frac{x}{4} + 0.5} \right)}}} \right)\frac{{t^{\alpha } }}{{{\Gamma }\left( {\alpha + 1} \right)}} \\ & \quad + \frac{1}{32768}\left( {sech^{4} \left( {\frac{x}{4} + 0.5} \right)\left\{ {\cosh \left( {\frac{x}{2} + 1} \right) - 2} \right\}} \right){ }\frac{{t^{2\alpha } }}{{{\Gamma }\left( {2\alpha + 1} \right)}} + \ldots \\ \end{aligned}$$

### Example 2

Consider time fractional CDGE (1) with initial condition

21 $$u\left( {x,0} \right) = \frac{{15 + \sqrt {105} }}{30} - tanh^{2} \left( x \right).$$

The exact solution in classical case is

22 $$u\left( {x,t} \right) = \frac{{15 + \sqrt {105} }}{30} - tanh^{2} \left( {x - 2\left( {11 - \sqrt {105} } \right)t} \right).$$

By using initial condition (21) and applying the steps of using LRPSM for solving the fractional CDGE which discussed in Section “Preliminaries”, we obtain

23 $$f_{0} \left( x \right) = u\left( {x,0} \right) = \frac{{15 + \sqrt {105} }}{30} - tanh^{2} \left( x \right),$$

24 $$f_{1} \left( x \right) = 3.0122\left( {\frac{\sinh \left( x \right)}{{cosh^{3} \left( x \right)}}} \right),$$

25 $$f_{2} \left( x \right) = \left( {9.0733sech^{2} \left( x \right) - 13.61sech^{4} \left( x \right)} \right).{ }$$

And the approximate solution fractional CDGE is:

26 $$\begin{aligned} u\left( {x,t} \right) & = \frac{{15 + \sqrt {105} }}{30} - tanh^{2} \left( x \right) + 3.0122\left( {\frac{\sinh \left( x \right)}{{cosh^{3} \left( x \right)}}} \right)\frac{{t^{\alpha } }}{{{\Gamma }\left( {\alpha + 1} \right)}} \\ & \quad + \left( {{9}.0{733}\;sech^{2} \left( x \right) - {13}.{61}\;sech^{4} \left( x \right)} \right){ }\frac{{t^{2\alpha } }}{{{\Gamma }\left( {2\alpha + 1} \right)}} \\ \end{aligned}$$

## Discussions and conclusion

**Figure 1:**
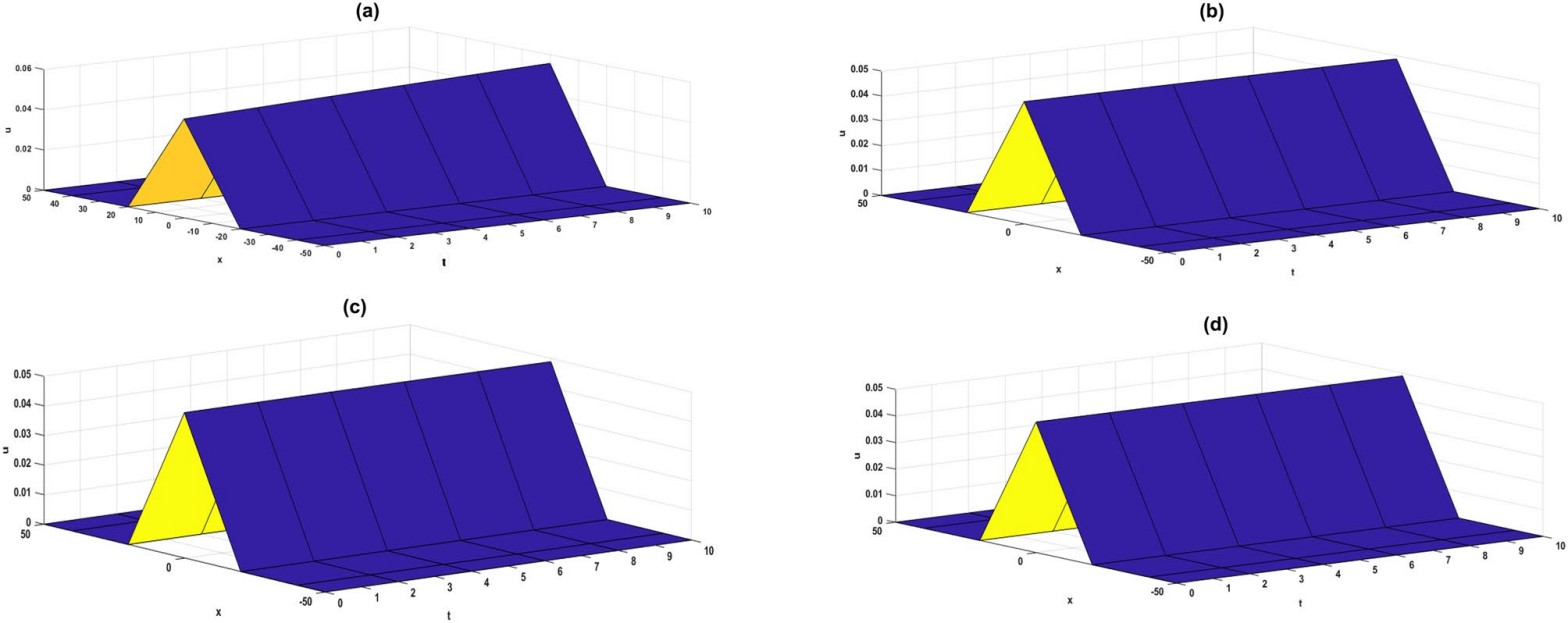
Numerical results for example 1 (**a**) Exact solution (**b**) $\alpha =1$ (**c**) $\alpha =0.9$ (**d**) $\alpha =0.8$

**Figure 2:**
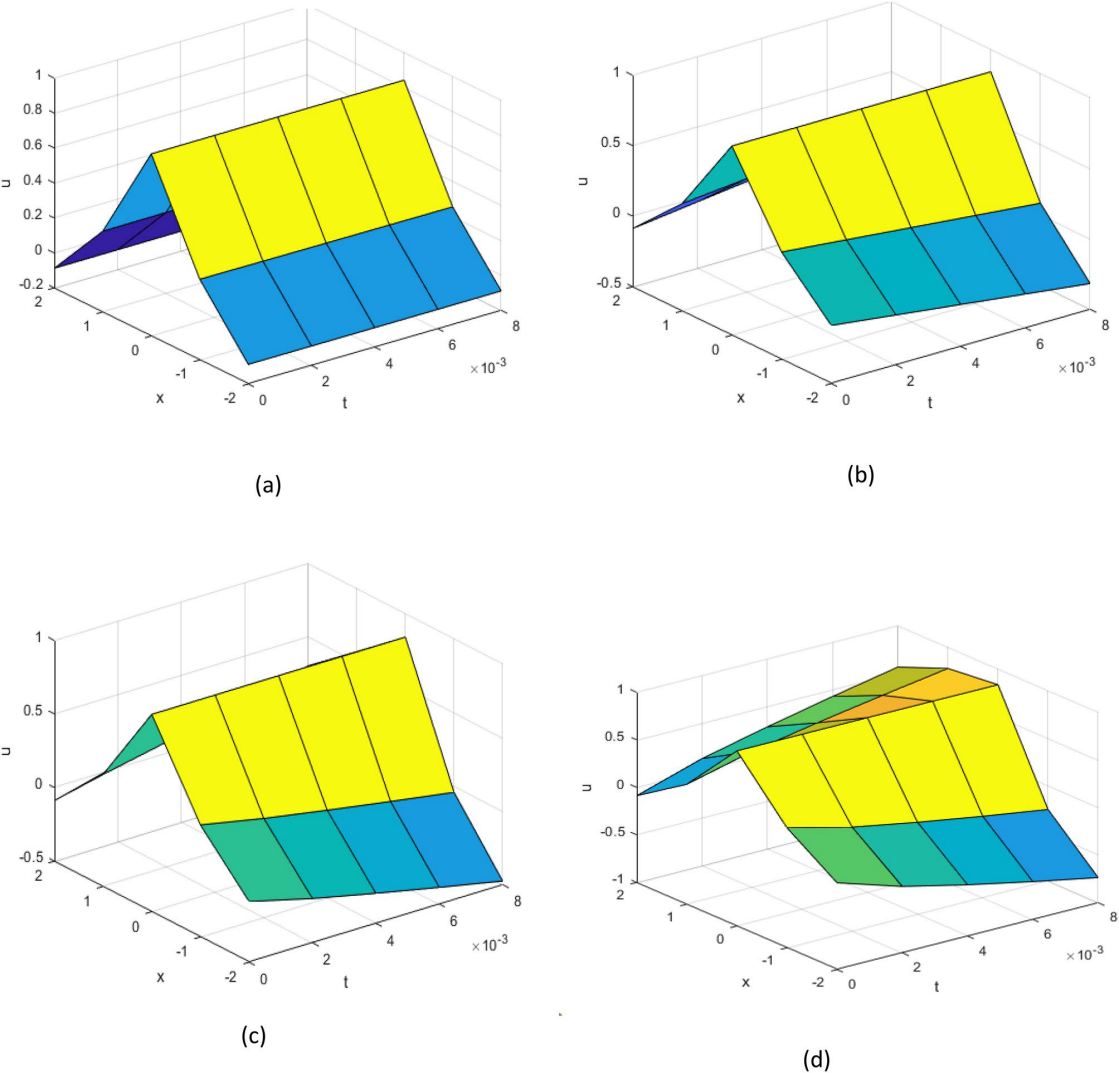
Numerical results for example 2 (**a**) Exact solution (**b**) $\alpha =1$ (**c**) $\alpha =0.9$ (**d**) $\alpha =0.8$

This paper introduces a series approximate solution to the fractional CDGE using LRPSM. For clarifying the accuracy and efficiency of the present method, the tables and graphs are shown the numerical results of such problems with the help of limit concept. A comparison was made between LRPSM, FTC-VIM, and FTC-HPM on example 1 in Table 1, and a comparison was also made between the LRPSM with NTDM and HASTM shown in Table 2 on example 2. From the two tables, it is proven that LRPSM is more accurate than the other methods. Figures 1 and 2 show the 3D-solutions for different initial value of the current problem to show the behaviour of LRPS solution at the different alpha values. It has been proven that the results are accuracy and efficiency with simplest way. We indicating that the LRPSM approach is one of the most effective ways to solve fractional order differential equations. In the near future, we look forward to use Laplace transform with other analytic method to achieve a high-accuracy solution with lower expansion terms.

**Table 1:** Comparison between LRPSM with FTC-VIM and FTC-HPM for example 1.

x	t	Numerical solution					
		LRPSM with 3 terms			LRPSM with 2 terms	FTC-VIM^37^	FTC-HPM^37^
		α = 0.7	α = 0.9	α = 1	α = 1	α = 1	α = 1
-50	0	0	0	0	0	0	0
	2	6.40972e−14	1.87107e−14	3.78100e−16	1.80551e−14	1.76778e−11	1.76778e−11
	4	3.04059e−13	1.07794e−13	2.97855e−15	7.07545e−14	3.53211e−11	3.53211e−11
	6	5.67030e−13	2.13785e−13	9.90035e−15	1.55999e−13	5.29318e−11	5.29318e−11
	8	8.32476e−13	3.27108e−13	2.31153e−14	2.71817e−13	7.05119e−11	7.05119e−11
	10	1.09255e−12	4.43976e−13	4.44757e−14	4.16356e−13	8.80633e−11	8.80633e−11
-40	0	0	0	0	0	0	0
	2	9.51288e−12	2.77692e−12	5.61150e−14	2.67962e−12	2.62363e−09	2.62363e−09
	4	4.51263e−11	1.59981e−11	4.42056e−13	1.05009e−11	5.24212e−09	5.24212e−09
	6	8.41547e−11	3.17285e−11	1.469342e−12	2.31523e−11	7.85578e−09	7.85578e−09
	8	1.23550e−10	4.85472e−11	3.43061e−12	4.03412e−11	1.04649e−08	1.04649e−08
	10	1.62149e−10	6.58920e−11	6.60078e−12	6.17927e−11	1.30697e−08	1.30697e−08
0	0	0	0	0	0	0	0
	2	1.50403e−04	4.18130e−05	1.26391e−06	1.85127e−05	6.54334e−02	6.54334e−02
	4	7.46909e−04	2.52505e−04	1.01620e−05	7.91573e−05	1.30909e−01	1.30909e−01
	6	1.41638e−03	4.96853e−04	3.44342e−05	1.89673e−04	1.96434e−01	1.96434e−01
	8	2.09931e−03	7.42150e−04	8.18630e−05	3.57844e−04	2.62018e−01	2.62018e−01
	10	2.76599e−03	9.68940e−04	1.60187e−04	5.91408e−04	3.27666e−01	3.27666e−01
40	0	0	0	0	0	0	0
	2	1.24156e−12	3.47519e−13	7.83542e−15	3.78077e−13	4.02445e−10	4.02445e−10
	4	6.99716e−12	2.45302e−12	6.36846e−14	1.54465e−12	8.04102e−10	8.04102e−10
	6	1.44110e−11	5.42196e−12	2.18401e−13	3.55058e−12	1.20491e−09	1.20491e−09
	8	2.3152e−11	9.15479e−12	5.26119e−13	6.44999e−12	1.60484e−09	1.60484e−09
	10	3.31208e−11	1.36464e−11	1.04446e−12	1.03005e−11	2.00381e−09	2.00381e−09
50	0	0	0	0	0	0	0
	2	8.36560e−15	2.34156e−15	5.27946e−17	2.54746e−15	2.71165e−12	2.71165e−12
	4	4.71465e−14	1.65283e−14	4.29103e−16	1.04077e−14	5.41799e−12	5.41799e−12
	6	9.71007e−14	3.65329e−14	1.47157e−15	2.39236e−14	8.11868e−12	8.11868e−12
	8	1.55998e−13	6.16844e−14	3.54496e−15	4.34597e−14	1.08133e−11	1.08133e−11
	10	2.23166e-13	9.19492e-14	7.03753e-15	6.94043e-14	1.35016e-11	1.35016e-11

**Table 2:** Comparison between LRPSM with ${\text{NTDM}}$ and HASTM for example 2.

x	t	Exact Solution	Numerical results			
			Present Method (LRPSM)	ERROR-LRPSM	ERROR-HASTM^36^	ERROR-${\text{NTDM}}$^38^
			α = 1	α = 1	α = 1	α = 1
0.5	0	0.6280127	0.628012	0	0	0
	0.01	0.6388936	0.638893	2.26691e−06	3.51211e−05	6.63723e−06
	0.02	0.6496327	0.649632	1.81241e−05	2.81021e−04	7.7452e−05
	0.03	0.6602163	0.6602163	6.12895e−05	9.48603e−04	1.38205e−04
	0.04	0.6706305	0.6706305	1.45589e−04	2.24888e−03	1.17e−03
	0.05	0.6808615	0.6808615	2.84925e−04	4.39294e−03	1.8875e−03
1	0	0.2615393	0.2615393	0	0	0
	0.01	0.2712439	0.27124439	3.99819e−07	1.41429e−04	7.0112e−06
	0.02	0.2810871	0.28109041	3.23987e−06	2.24888e−03	2.78788e−05
	0.03	0.2910662	0.29107745	1.12342e−05	3.81193e−04	1.2331e−04
	0.04	0.3011780	0.30120548	2.74038e−05	9.02767e−04	1.1006e−03
	0.05	0.311419	0.31147452	5.50858e−05	1.76163e−03	1.0116e−03

## Data Availability

All data generated or analysed during this study are included in this published article.
